# First Report of Chromosome-Level Genome Assembly for Flathead Grey Mullet, *Mugil cephalus* (Linnaeus, 1758)

**DOI:** 10.3389/fgene.2022.911446

**Published:** 2022-06-17

**Authors:** Mudagandur S. Shekhar, Vinaya Kumar Katneni, Ashok Kumar Jangam, Karthic Krishnan, Sudheesh K. Prabhudas, Jesudhas Raymond Jani Angel, Krishna Sukumaran, Muniyandi Kailasam, Joykrushna Jena

**Affiliations:** ^1^ Nutrition Genetics and Biotechnology Division, Indian Council of Agricultural Research-Central Institute of Brackishwater Aquaculture, Chennai, India; ^2^ Finfish Culture Division, ICAR-Central Institute of Brackishwater Aquaculture, Chennai, India; ^3^ Indian Council of Agricultural Research, New Delhi, India

**Keywords:** whole genome, repeat analysis, synteny, grey mullet, PacBio Sequel II, genome annotation

## Introduction

Amid dwindling production from capture fisheries, aquaculture is an important source of high-quality protein (FAO Food and Agriculture Organization of the United Nations, 2020). In addition, aquaculture generates employment and contributes to eradicating poverty. *Mugil cephalus*, flathead grey mullet, is globally distributed and inhabits inshore seas, estuaries, and brackish water areas (Whitfield et al., 2012). It is of important commercial value to global fisheries and aquaculture with high demand for mullet roe. This species belongs to the family Mugilidae which comprises 26 genera and 80 valid species (Fricke et al., 2022). The adaptability of *M. cephalus* to varied aquatic environments at different life stages and tolerance to a wide range of salinities and temperatures occurring in tropical, subtropical, and temperate coastal waters make it an important cultivatable fish species across the world. The whole-genome sequence information for an aquaculture species has potential applications in genomic selection and selective breeding for sustainable production and improvement of desirable traits, such as disease resistance and growth.

Within the family Mugilidae, a draft genome assembly was first reported for redlip mullet, *Liza haematocheila* (0.74 Gb), which has 1,453 contigs with an N50 length of 3.9 Mb (Liyanage et al., 2019). Later, chromosome-level assembly of this fish was generated with Oxford Nanopore long-read, single-tube long fragment reads (stLFR), and HiC chromatin interaction data, which are 652.91 Mb length in 514 scaffolds with contig and scaffold N50 lengths of 7.21 and 28.01 Mb, respectively (Zhao et al., 2021). For *M. cephalus*, no whole-genome assembly with long-read data is available. Previous reports have attempted genome assembly of this fish at a very low sequence depth using Illumina technology (Dor et al., 2016, 2020). Thus, in the absence of a reference genome for *M. cephalus*, this study aimed to decipher the whole-genome sequence, which will provide baseline information needed to implement genetic improvement programs. The integration of genome information into fisheries and aquaculture management is important to ensure long-term sustainable fishery harvest and aquaculture production. In the present study, a combination of PacBio, Illumina, and Arima Hi-C technologies were applied to construct the genome assembly of *M. cephalus*, an economically important brackish water aquaculture species.

### Value of Data

– The whole-genome sequence assembly generated for *M. cephalus* can be used as a reference genome for the family Mugilidae.
– The high-quality, chromosome-level genome assembly along with the predicted protein sequences would help gain insights into desirable traits through gene expression studies.
– The whole-genome assembly provides baseline information needed to implement genetic improvement programs for this commercially important species.

## Materials and Methods

### Specimen of *M. cephalus*

A specimen of *M. cephalus* maintained at the Muthukadu Experimental Station of ICAR—CIBA (Chennai, India) was used for generating the sequence data required for genome assembly. The species identity of the specimen was confirmed based on the partial sequence of the barcode gene, cytochrome C oxidase I (COI). Briefly, genomic DNA was isolated using the conventional CTAB method (Mirimin and Roodt-Wilding, 2015) from muscle tissue, and its concentration and quality were assessed using a nanodrop 2000C (Thermo Scientific, Waltham, Massachusetts, United States). A partial fragment of the COI gene was amplified using high-fidelity 2X PCR Master Mix (New England Biolabs, Ipswich, Massachusetts, United States) with the primers, F2 - 5′TCG​ACT​AAT​CAC​AAA​GAC​ATC​GGC​AC3′ and R1 - 5′TAG​ACT​TCT​GGG​TGG​CCA​AAG​AAT​CA3′ (Ward et al., 2005). The amplification conditions were initial denaturation at 98°C for 30 s; 32 cycles of denaturation at 98°C for 10 s, annealing at 55°C for 30 s, and extension at 72°C for 30 s; followed by a final extension at 72°C for 2 min. The PCR product of 707 bp was gel-extracted using a QIAquick gel extraction kit (Qiagen, Hilden, Germany) and sequenced bidirectionally using an ABI 3730 sequencer (Applied Biosystems, Waltham, Massachusetts, United States). The partial sequence of the COI gene was submitted to GenBank with accession number, MW584357. The sequence of MW584357 was subjected to phylogenetic analysis along with 476 accessions of the genus *Mugil* (Supplementary Table S1) sourced from the Barcode of Life Data systems database^1^ and accession of *Chelon labrosus* as an outgroup. Initially, all the 478 sequences were aligned in BioEdit version 7.2.5 (Hall, 1999) to generate a consensus alignment (516 bp) which was used to build a Maximum Likelihood tree in RAxML version 8.2.9 (Stamatakis, 2014) with the GTRGAMMAI model and a random seed value of 12,345. The final tree was visualized using FigTree v1.4.3^2^, which revealed the clustering of sequence MW584357 with other accessions of *M. cephalus* (Supplementary Figure S1).

### PacBio Sequel II Data Generation

Genomic DNA was extracted from the muscle tissue of *M. cephalus* using the blood and cell culture DNA midi kit (Qiagen). DNA quantification was carried out using a Qubit 4.0 fluorometer (Thermofisher Scientific). Library preparation was performed with the SMRTbell^®^ Express Template Prep Kit 2.0 (Pacific Biosciences, Menlo Park, California, United States), and size selection was carried out using BluePippin™ (Sage Science, Beverly, Massachusetts, United States). The library was sequenced on the PacBio Sequel II platform (Pacific Biosciences) to generate the sequence data. About 257.7 Gb of sequence data in 15,028,480 subreads was generated with a subread N50 of 28,748 bp (Supplementary Table S2).

### Illumina Data Generation

Genomic DNA extracted from the muscle tissue of *M. cephalus* was used for Illumina library preparation with an insert size of 200–300 bp using the NEBNext^®^ Ultra™ II DNA Library Prep Kit for Illumina^®^ (New England Biolabs). The PCR products used for the construction of the library were purified with the AMPure XP reagent (Beckman Coulter, Brea, California, United States). The library was checked for size distribution by Agilent 2,100 Bioanalyzer (Agilent Technologies, Santa Clara, California, United States) and sequenced on Illumina NovaSeq 6,000, S4 Flow Cell (2 × 150 bp read length). About 640 million reads/96.1 Gb data were generated with 93% of Q30 bases (Supplementary Table S3). The Illumina paired-end reads were used to assess genome length and also to correct the erroneous bases in genome assembly contigs.

### HiC Data Generation

For HiC data generation, tissue crosslinking and proximity ligation were performed using the Phase Genomics Kit (Phase Genomics, Seattle, Washington, United States) followed by Illumina compatible sequencing library preparation. The library was sequenced on the Illumina NovaSeq6000 platform in a 150-bp paired-end mode to generate 181 million paired reads (54.31 Gb), of which 90.74% (49.28 Gb) of bases had a Q30 quality score (Supplementary Table S4). The HiC reads were used in the scaffolding of assembly contigs.

### Genome Length Assessment

The *k*-mer frequency for the grey mullet genome was estimated from the Illumina paired-end reads using Jellyfish 2.2.3 (Marçais and Kingsford, 2011) to count the canonical 21 *k*-mers with the hash size as 20G. The count histogram was later provided as the input to the online tool Genomescope (Vurture et al., 2017) to estimate genome haploid length, heterozygosity, and repeat content. The *k*-mer analysis revealed the genome haploid length to be 594 Mb and genome repeat length as 48 Mb (Supplementary Figure S2). The genome size of *M. cephalus* was reported to be 857 Mb based on the flow cytometry principle (Raymond et al., 2022).

### Genome Assembly

Genome assembly was performed with the WTDBG2.5 (Ruan and Li, 2020) tool by limiting the usage of PacBio subreads to >30 kb length covering 100 Gb of data. The *de novo* contig-level assembly contained 848 contigs with an N50 length of 20.15 Mb. Polishing of these contigs for base errors and indels with Illumina short reads using POLCA (Zimin and Salzberg, 2020) brought the base consensus quality to 99.99% (Supplementary Table S5). Scaffolding was performed on the polished assembly by using juicer and 3D-DNA scripts from the Genome Assembly Cookbook (Dudchenko et al., 2017). Initially, site positions were generated based on the DpnII enzyme used for generating 181 million HiC read pairs Later, a juicer script was used to generate contact maps in 3D space, and a 3D-DNA script was used to anchor fragments to pseudo-chromosomes which were visualized in Juicebox v1.11.08 (Supplementary Figure S3).

**FIGURE 1:**
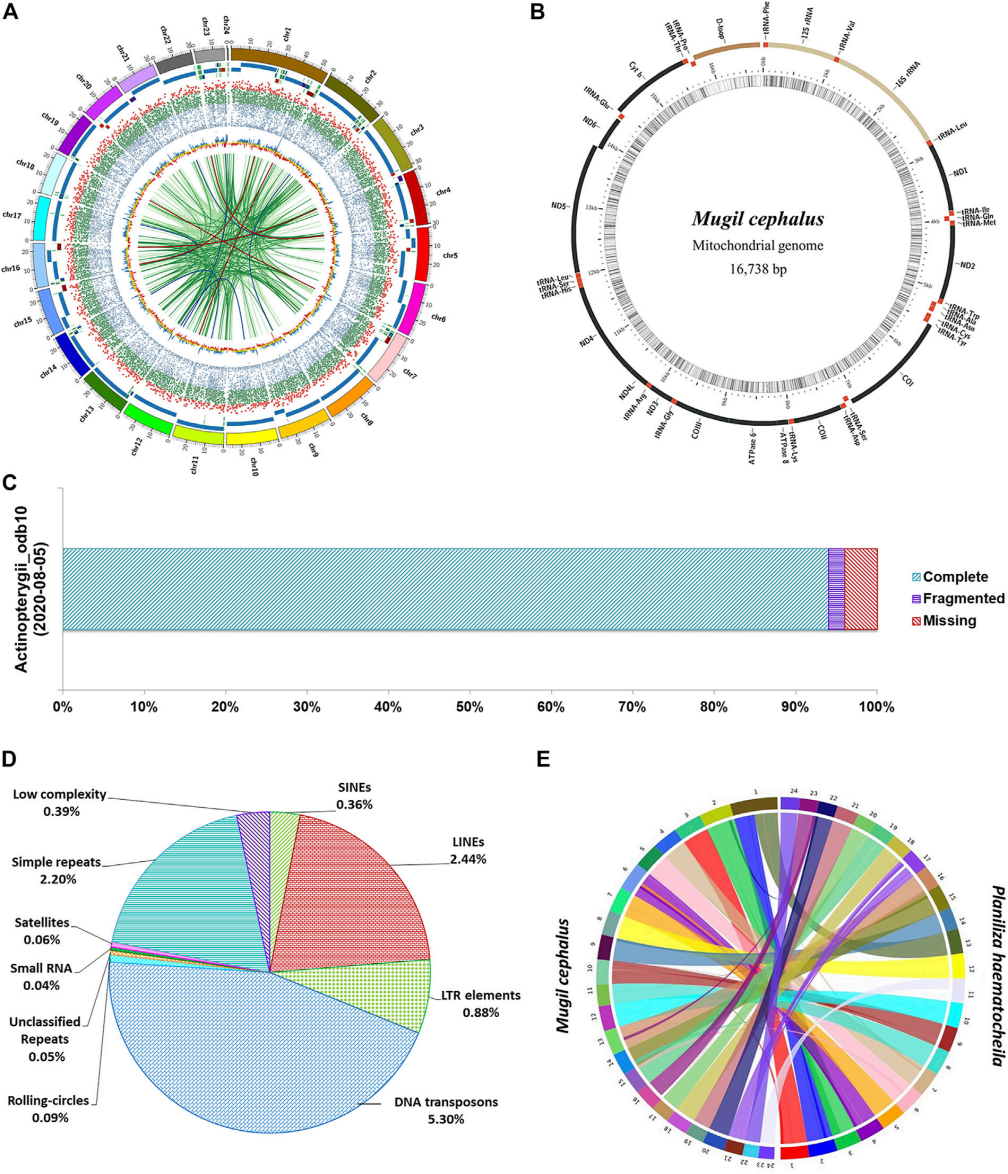
Assembly of the *M. cephalus* genome. **(A)** Circos plot depicting the 24 pseudo-chromosomes in the *M. cephalus* assembly and their features. The track 1 (outermost) depicts 24 pseudo-chromosomes; track 2 depicts contigs represented as tiles (contigs <5 Mb are shown in variable colors); track 3 shows protein-coding genes as a scatter plot (gene length of <5 kb in blue, 5–30 kb in green >30 kb in red); track 4 shows GC content of the genome as a yellow line plot (GC values >43 in blue and <41 in red); track 5 (innermost) depicts self synteny in the genome shown as a link plot (alignment lengths over 1,500 bp in light green, 2–3 kb in dark green, 3–3.5 kb in dark blue and >3.5 kb in dark red). **(B)** Map of the complete mitochondrial DNA genome of *M. cephalus*. **(C)** BUSCO scores illustrating the completeness of the *M. cephalus* genome assembly. **(D)** Profile of repetitive elements in the *M. cephalus* genome assembly. **(E)** Synteny plot between redlip mullet and grey mullet genome assemblies.

At the scaffold level, the assembly is 644 Mb in length in 583 scaffolds with an N50 of 28.32 Mb. The *M. cephlus* karyotype is reported to contain 24 chromosome pairs (Gornung et al., 2001). Accordingly, the longest 24 scaffolds represented 98.56% (634 Mb) of the scaffold-level assembly length and hence are designated as pseudo-chromosomes (Table 1). The mitochondrial genome of *Mugil cephalus* was obtained as a single scaffold of 16,738 bp in the assembly. The mitochondrial genome was annotated (Figure 1B) using the MitoAnnotator tool^3^ (Iwasaki et al., 2013). The genome assembly presented for *M. cephalus* in this study is superior to the assembly reported by Dor et al. (2016) with 480,389 scaffolds and to that reported by Dor et al. (2020) with 4,505 scaffolds in terms of N50 statistics and average scaffold length. The genome assembly reported in this study is of a shorter length than the estimated genome length reported in previous studies (Hinegardner and Rosen, 1972; Dor et al., 2016, 2020; Raymond et al., 2022). The genome completeness for *M. cephalus* assembly was assessed to be 96% complete (Figure 1C) using BUSCO v5.2.2 (Seppey et al., 2019) against the Actinopterygii_odb10 (2020-08-05) orthologous dataset (Kriventseva et al., 2019).

**TABLE 1:** Summary of *Mugil cephalus* genome assembly and annotation.

Features	Statistics
No. of contigs	848
Contig N50 size (Mbp)	20.15
Contig L50	14
Contig total length (Mbp)	643.89
No. of scaffolds	583
Scaffold N50 size (Mbp)	28.32
Scaffold L50	10
Total scaffold length (Mbp)	644.11
Number of pseudo-chromosomes	24
Total length of pseudo-chromosomes (Mbp)	634.85
GC %	41.9
No. of protein coding genes	27,269

### Repeat Prediction

Repeat masking was performed by using the Repeat masker v4.0.9^4^ available in the genome analysis module of Omicsbox v2.0.36 (Bioinformatics, 2019). The RMBlast search was performed for 24 pseudo-chromosomes with a total of 634,849,760 bp against the Eukaryota subset of the RepBase Repeat Masker Edition-20181026^5^. The interspersed, low-complexity, and simple-sequence repeats were masked to obtain the complete repeat profile of *M. cephalus* genome assembly. The total repetitive elements in *M. cephalus* assembly constitute 11.72% (74,376,509 bp) of assembly length as shown in Supplementary Table S6. DNA transposons (5.3%, 33,664,198 bp) and the satellite repeats (0.06%, 362,353 bp) contributed the highest and the lowest, respectively, to the total repeat content of the genome (Figure 1D). The LINEs were the major contributor for retroelements, followed by LTR elements and SINEs accounting for 2.44, 0.88, and 0.36%, respectively. Simple-sequence repeats and low-complexity repeats covered 2.2% (13,966,215 bp) and 0.39% (2,504,867 bp) of the genome, respectively. The MISA v1.0 tool was used to generate the simple sequence repeat profile containing a total of 491,676 SSR elements in assembly (Supplementary Figure S4).

### Gene Prediction

The pseudo-chromosomes soft-masked for interspersed sequence repeats avoiding the low-complexity and simple-sequence repeats were used for gene prediction. Structural annotation of the *Mugil cephalus* genome was performed by following the approach as described previously (Katneni et al., 2022), with minor modifications. A combination of *ab initio* and evidence-based approaches was used to obtain the final protein-coding gene set. Briefly, *ab initio* prediction was performed using Augustus v3.3.3 (Stanke et al., 2006) and GeneMark-ES v4.59 (Lomsadze et al., 2005) on the repeat-masked genome. The Illumina paired-end RNA-seq data of fish tissues and developmental stages accessed from GenBank (SRR13039561-66; SRR15243984-88 and SRR16311547-49) were mapped to the genome assembly using Hisat2 v2.2.0 (Kim et al., 2019) aligner. Then, StringTie v2.1.4 (Pertea et al., 2015) was used on this sorted alignment file to assemble the transcripts which were processed in TransDecoder v5.5.0^6^ to obtain the Open-Reading Frames (ORFs). Similarly, PacBio IsoSequence reads (NCBI Bioproject: PRJNA675305) were aligned to the genome assembly using GMAP v2020-06-30 (Wu and Watanabe, 2005) to generate valid gene structures using PASA v2.4.1 (Haas et al., 2003) and then finally to generate genome coordinates using TransDecoder v5.5.0^6^. Furthermore, evidence was gathered from the proteins of related species (*Archocentrus centrachus*, *Astatotilapia calliptera*, *Gambusia affinis*, *Melanotaenia boesemani*, *Maylandia zebra*, *Oreochromis niloticus*, *Oryzias melastigma*, *Parambassis ranga*, *Salarias fasciatus*, and *Xiphophorus maculatus* genome assemblies) by aligning them to the *M. cephalus* genome using GenomeThreader v1.7.3 (Gremme, 2012). Finally, all the evidence from *ab initio* gene predictions, Isosequence reads, RNA-seq reads, and protein data of related species were combined using Evidence Modeler (Haas et al., 2008) to obtain the consensus and non-redundant protein-coding gene set containing 30,882 genes. Then, AGAT^7^ was used to filter out the incomplete gene models, resulting in the final gene set of 27,269 genes (Supplementary Table S7). Noncoding genes in the *M. cephalus* genome were identified by aligning the repeat-masked assembly to the Rfam^8^ database using infernal v1.1.4 (Nawrocki and Eddy, 2013). A total of 5,165 non-coding RNAs were identified (Supplementary Table S8), out of which 5S_RNA, tRNA, and histone3 were in abundance.

### Functional Annotation

Homology-based genome annotation was performed with the blastx tool (Altschul et al., 1990) using the Actinopterygii (txid7898) non-redundant database of GenBank. The protein domains and orthology-based annotations were executed by the Interproscan module and EggNOG mapper module (Huerta-Cepas et al., 2019), as implemented in OmicsBox v2.0.36 (Bioinformatics, 2019), respectively. Then, all the annotations were merged using OmicsBox v2.0.36 (Bioinformatics, 2019) to obtain the gene ontology of the annotated genes and map the enzyme codes. The pathway details of the annotated genes were obtained by mapping against the KEGG database (Kanehisa et al., 2017). Blastx hits were obtained for 22,334 (81.9%) transcripts from which 18,806 (69%) were functionally annotated and 8,113 (29.8%) were mapped with enzyme codes (Supplementary Figure S5). The transferases and hydrolases were the most dominant enzyme classes expressed (Supplementary Figure S6). Gene ontology analysis revealed that the most expressed GO categories were gene expression (Biological processes), metal ion binding (Molecular function), and intracellular membrane-bounded organelle (Cellular components) in *M. cephalus* proteins (Supplementary Figure S7).

### Synteny Analysis Between *Mugil cephalus* and *Planiliza haematocheila*

Comparative genome analysis was performed to characterize genome-wide syntenic regions between redlip mullet (Zhao et al., 2021) and grey mullet (this study) genomes. The 24 chromosome–level scaffolds of the redlip mullet assembly (648.41 Mb) obtained from the China National Gene Bank Database (CNGBdb: CNP0001604) were aligned to the 24 pseudo-chromosomes of the grey mullet assembly (634.84 Mb) using SyMAP 4.2 (Soderlund et al., 2006). A total of 93 synteny blocks were observed between the two genomes. Among them, 24 blocks of the grey mullet genome were found to align against 25 blocks of the redlip mullet genome with block lengths greater than 10 Mb (Figure 1E; Supplementary Table S9).

## Data Availability

The datasets presented in this study can be found in online repositories. The names of the repository/repositories and accession number(s) can be found below: https://www.ncbi.nlm.nih.gov/, PRJNA675305; https://figshare.com/, https://doi.org/10.6084/m9.figshare.19499054.v1.

## Footnotes

1
www.boldsystems.org

2
http://tree.bio.ed.ac.uk/software/figtree/

3
http://mitofish.aori.u-tokyo.ac.jp/annotation/input.html

4
http://www.repeatmasker.org/

5
https://www.girinst.org/repbase/

6
https://github.com/TransDecoder

7
https://github.com/NBISweden/AGAT

8
http://rfam.xfam.org/
