# Supplementary material for: First Report of Chromosome-Level Genome Assembly for Flathead Grey Mullet, Mugil cephalus (Linnaeus, 1758)
Source: Front Genet. 2022 Jun 17;13:911446. doi: 10.3389/fgene.2022.911446 (PMC9247318; doi:10.3389/fgene.2022.911446)
Supplement: Supplementary file 1 [file DataSheet1.docx]

**Supplementary Figures**


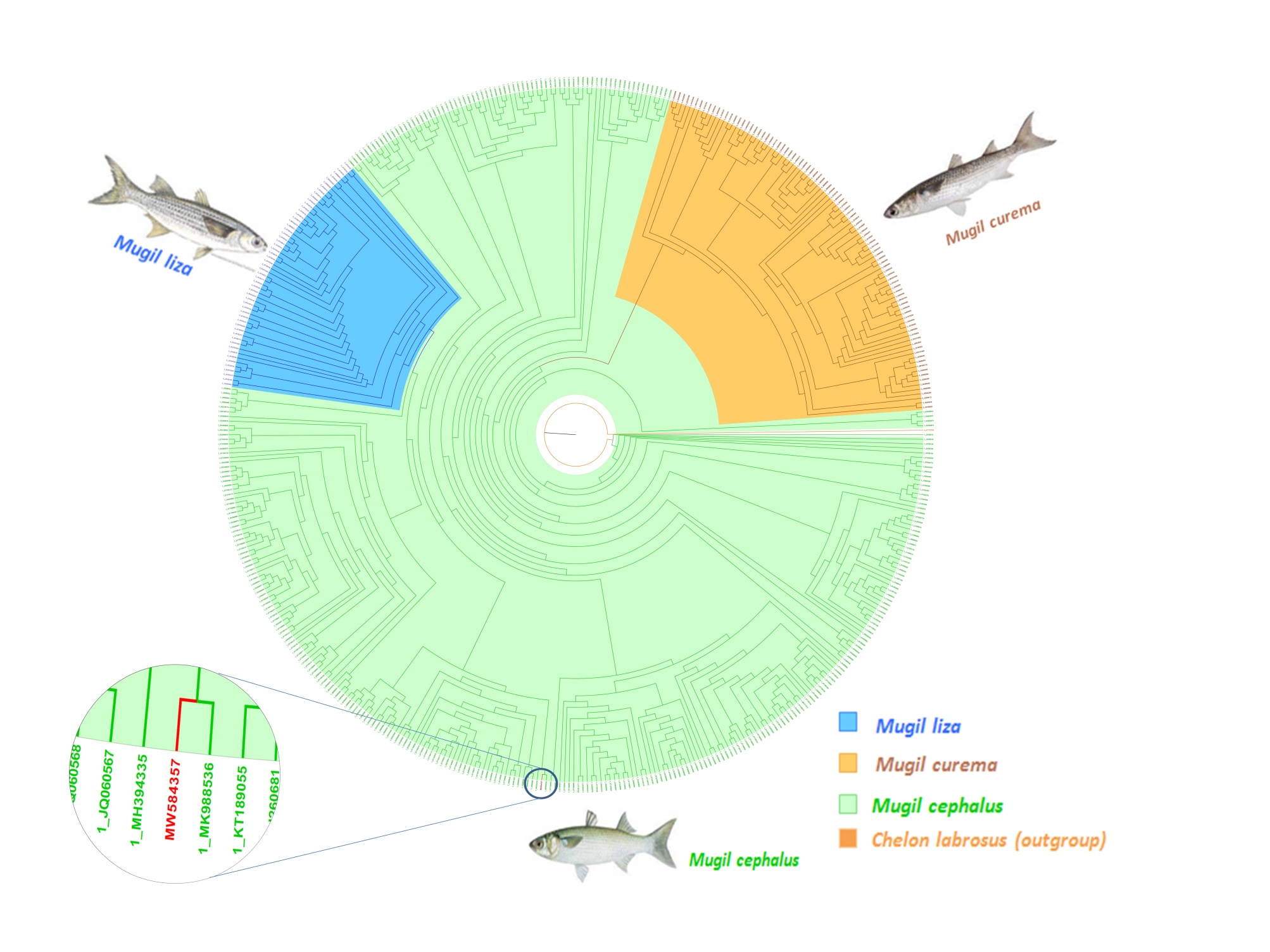


**Supplementary Figure 1:** Phylogenetic tree generated for the study specimen along with other Mugilid accessions based on Cytochrome C Oxidase I (COI) gene sequence.

**
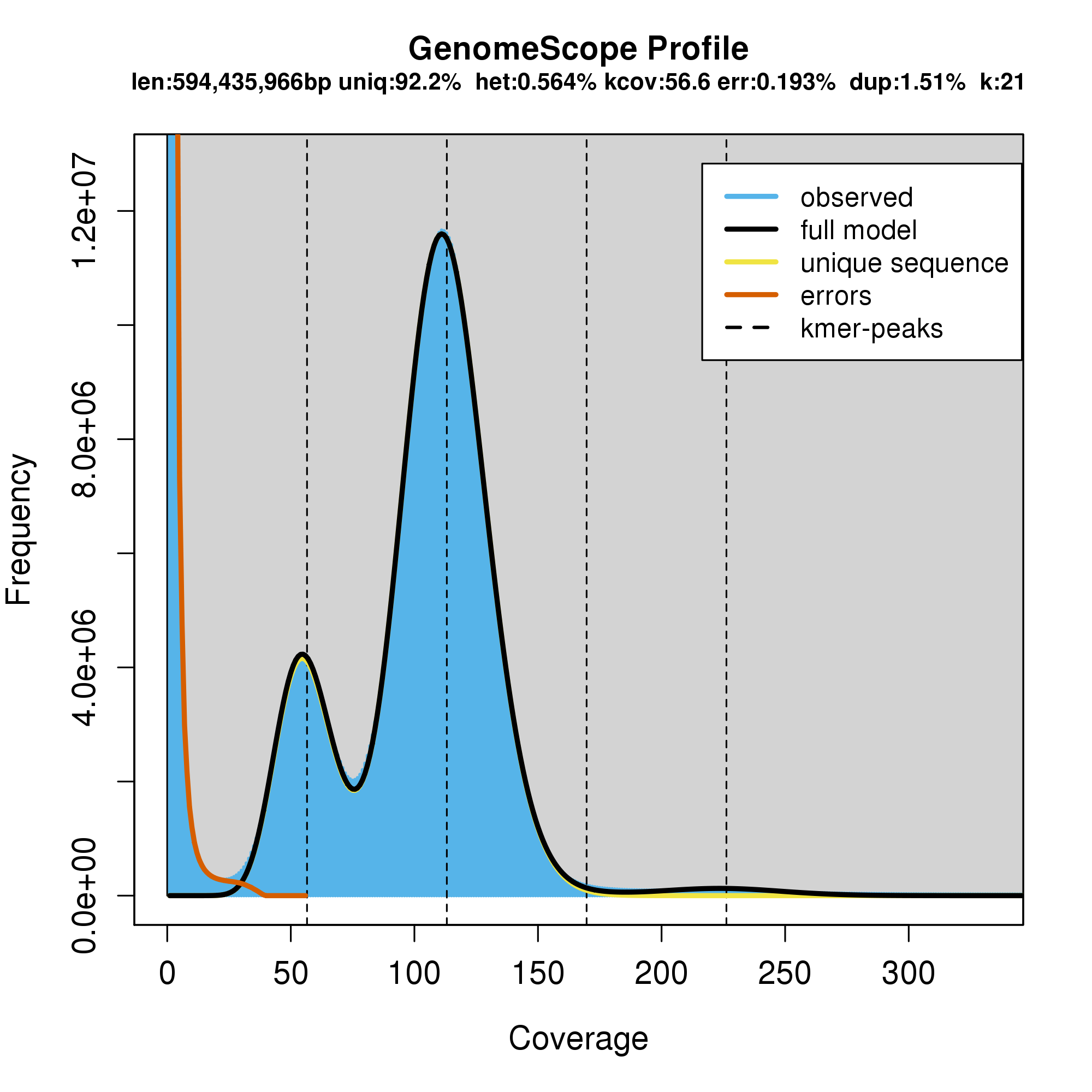
**

**Supplementary Figure 2:** Estimated genome size for *Mugil cephalus* genome based on k-mer profile using a mer size of 21.


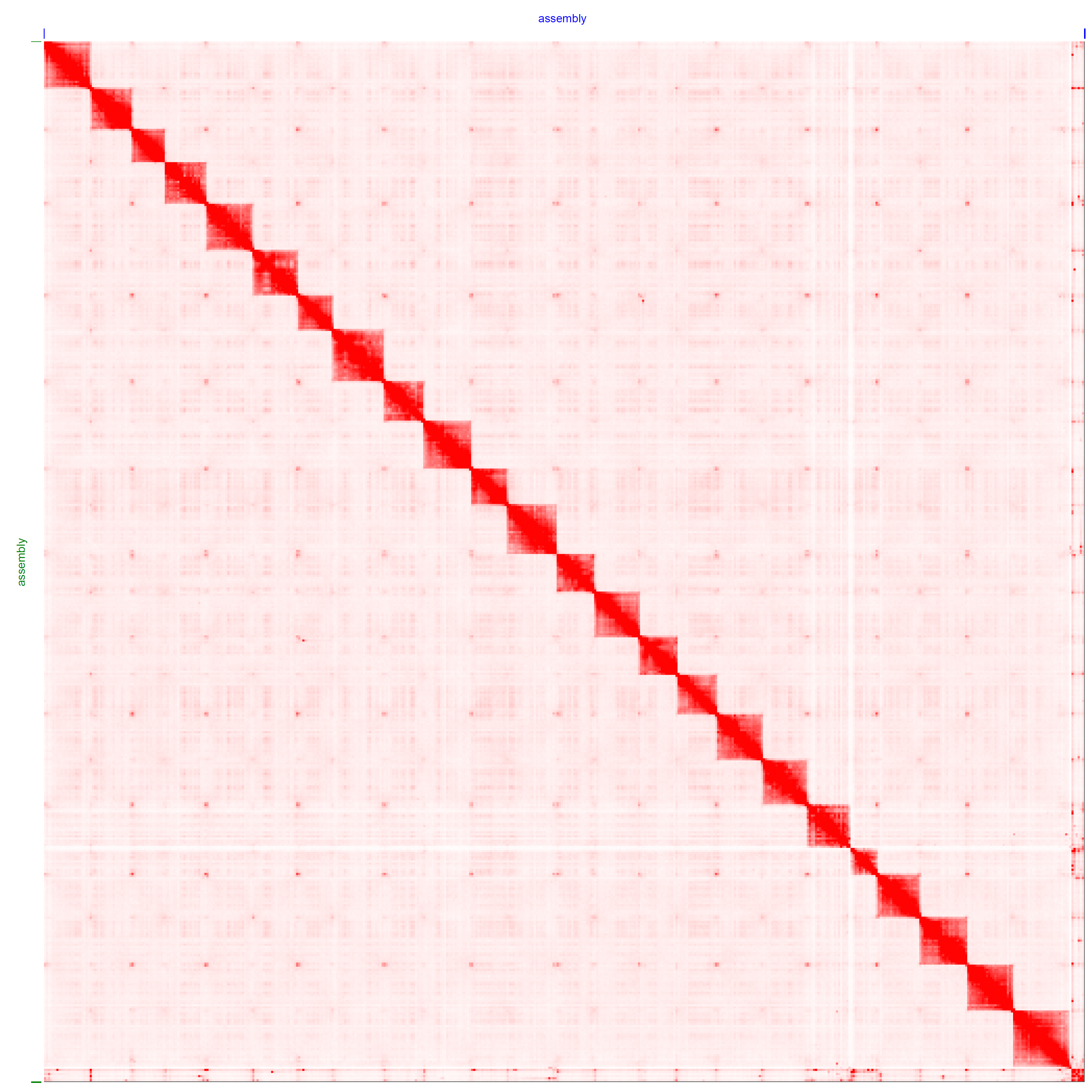


**Supplementary Figure 3:** Visualization of 24 pseudochromosomes of *Mugil cephalus* genome assembly in juicebox tool.

**Supplementary Figure 4: SSR Profile of *Mugil cephalus* genome assembly.** The bar plot indicates the number of SSRs in each SSR type and the red line indicates the percentage contributed by each type to the total SSRs.


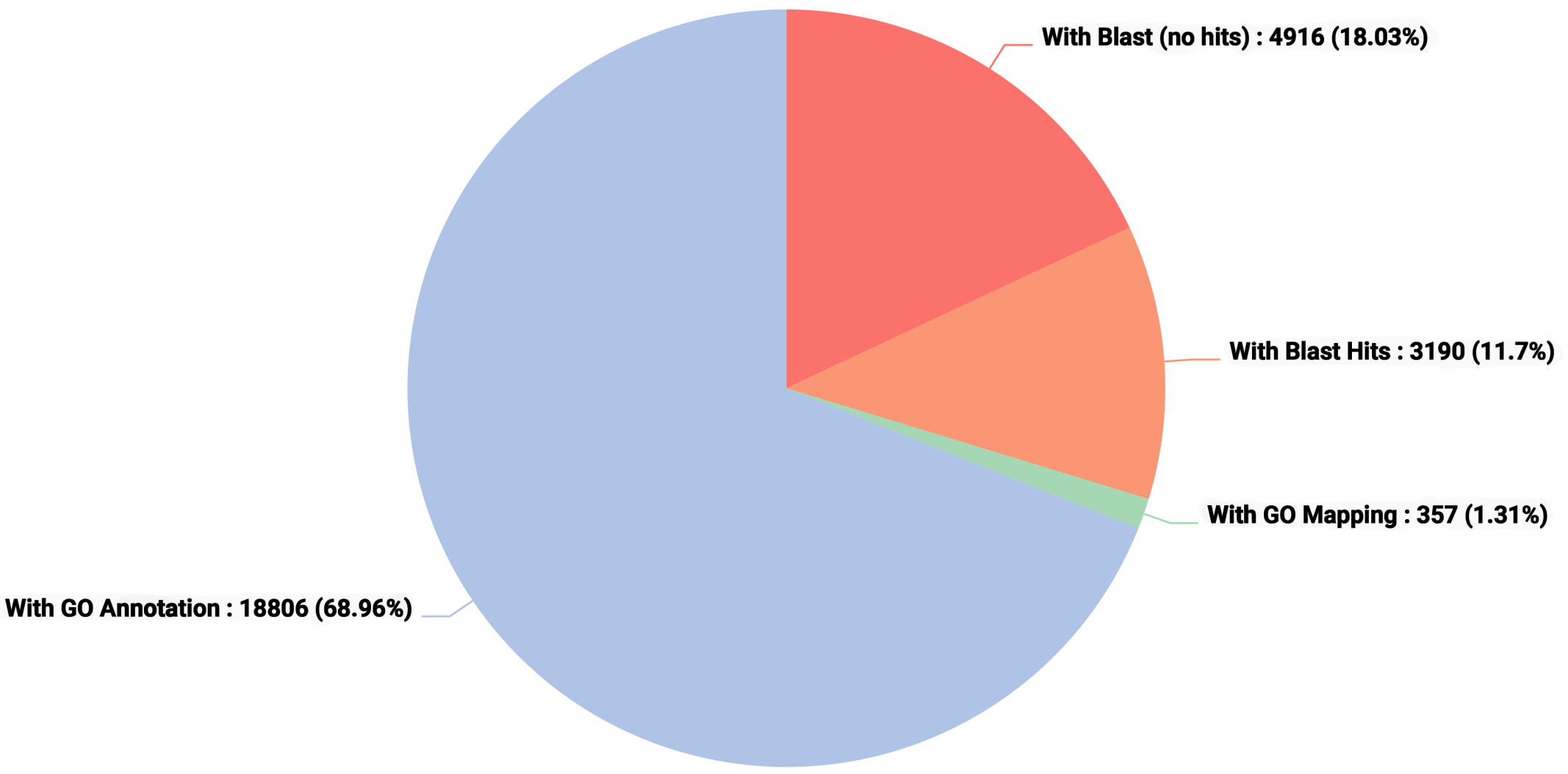


**Supplementary Figure 5:** Annotation summary of *Mugil cephalus* genome.


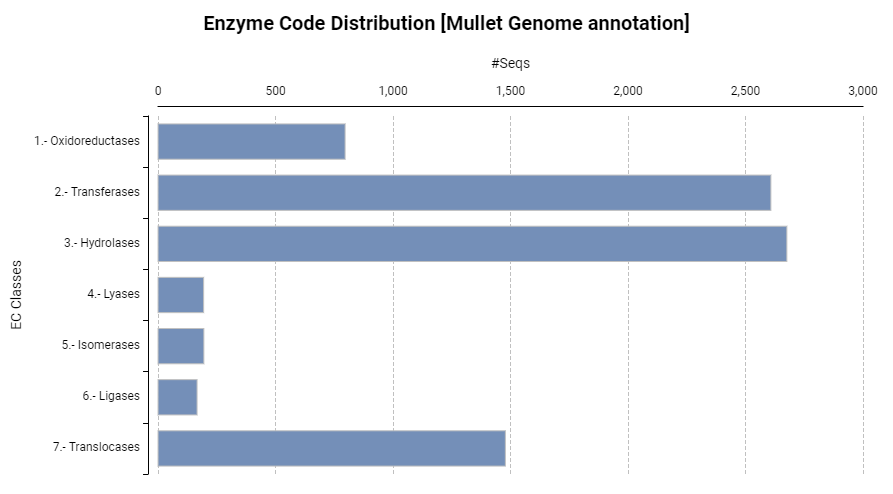


**Supplementary Figure 6:** Enzyme code distribution for the predicted protein-coding genes in *Mugil cephalus* genome assembly.


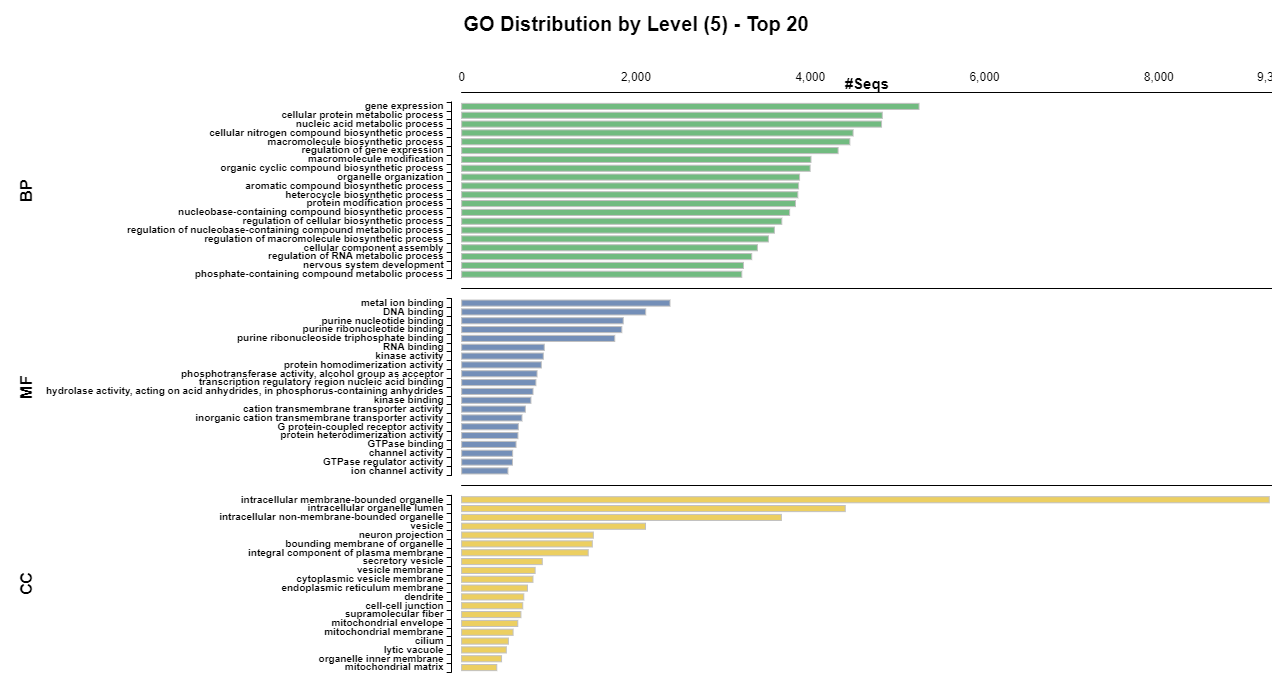


**Supplementary Figure 7:** Gene ontology distribution (by level 5) for the predicted protein-coding genes in *Mugil cephalus* genome assembly.

**Supplementary Tables**

**Supplementary Table 1:** The COI gene sequence accessions used for phylogenetic analysis in the study.

| **Database** | **Species** | **Number of accessions** | **Accession Numbers** |
| --- | --- | --- | --- |
| BOLD database | *Mugil cephalus* | 327 | AP002930, EU595084, EU595085, GU224562, GU224563, GU225391, GU225392, GU225393, GU225394, GU225395, GU260664, GU260665, GU260666, GU260667, GU260668, GU260669, GU260670, GU260671, GU260672, GU260673, GU260674, GU260675, GU260676, GU260677, GU260678, GU260679, GU260680, GU260681, GU260682, GU260683, GU260684, GU260685, GU260686, GU260687, GU260688, GU260689, GU260690, GU260691, GU260692, GU260693, GU260694, GU260695, GU260696, GU260697, GU672700, GU672701, GU672702, HM006970, HM208844, HM208845, HM208847, HM208848, HM208849, HM208850, HQ024977, HQ024978, HQ024979, HQ024980, HQ131884, HQ131885, HQ149082, HQ149083, HQ149710, HQ149714, HQ149715, HQ573262, HQ573280, HQ573281, HQ575807, HQ575808, HQ575809, HQ575811, HQ575822, HQ575823, HQ575824, HQ575825, HQ575826, JF493905, JF493906, JF493907, JN242565, JN242566, JN242567, JN242568, JN242569, JN242570, JN242571, JQ045783, JQ060528, JQ060529, JQ060530, JQ060531, JQ060532, JQ060533, JQ060534, JQ060535, JQ060536, JQ060537, JQ060538, JQ060539, JQ060540, JQ060541, JQ060542, JQ060544, JQ060545, JQ060546, JQ060547, JQ060548, JQ060549, JQ060550, JQ060551, JQ060552, JQ060553, JQ060554, JQ060555, JQ060556, JQ060557, JQ060558, JQ060559, JQ060560, JQ060561, JQ060562, JQ060563, JQ060564, JQ060565, JQ060566, JQ060567, JQ060568, JQ060569, JQ060570, JQ060571, JQ060572, JQ072904, JQ623956, JQ842582, JQ842583, JQ842584, JQ842585, JQ842586, JQ842587, JX185135, JX185136, JX185137, JX185138, JX185139, JX559532, JX559533, JX983367, JX983368, JX983369, KC500933, KC500934, KC500935, KC500936, KC500937, KC500938, KC500939, KC500940, KC500941, KC500942, KC500943, KC500944, KC500945, KC500946, KC500947, KC500948, KC500949, KC500950, KC500951, KC500952, KF930146, KJ552298, KJ553709, KJ553734, KJ553812, KJ553862, KJ553892, KJ553924, KJ553935, KJ669536, KJ669537, KM368340, KP018403, KP200024, KP856770, KP861225, KT189047, KT189048, KT189049, KT189050, KT189051, KT189052, KT189053, KT189054, KT189055, KT189056, KT189057, KT189058, KT189059, KT189060, KT189061, KT189062, KT189063, KT189064, KT189065, KT189066, KT189067, KT189068, KT189069, KT189070, KT189071, KT189072, KT189073, KT189074, KT189075, KT189076, KT189077, KT189078, KT189079, KT189080, KT189081, KT189082, KT189083, KT189084, KT189085, KT189086, KT189087, KT189088, KT189089, KT189090, KT189091, KT189092, KT189093, KT189094, KT189095, KT189096, KT189097, KT189098, KT189099, KT189100, KT189101, KT189102, KT189103, KT189104, KT189105, KT189106, KT189107, KT189108, KT189109, KT189110, KT189111, KT189112, KT189113, KT189114, KT189115, KT189116, KT189117, KT189118, KT189119, KT189120, KT189121, KT189122, KT189123, KT189124, KT189125, KT189126, KT189127, KT189128, KT189129, KT189130, KT189131, KT189132, KT189133, KT347596, KT347597, KT347598, KU892797, KU943211, KU943212, KU943213, KU943214, KU943226, KX639485, KX639486, KX639487, KX639488, KX781851, KX929971, KX929972, KX929973, KX929974, KY176531, KY652973, KY683176, KY780117, KY780118, KY849536, LC114159, LC424361, LC424362, LC424363, LC424364, LC424365, LC424366, LC424367, LC424368, LC424369, LC424370, LC487184, LC487185, LC487186, LC487187, LC487188, LC490608, LC490609, LC490611, LC490612, LC490613, LC490614, LC490615, MF098688, MG816703, MH394335, MK777473, MK988536, MN243481, MT888997. |
| BOLD database | *Mugil liza* | 56 | EU074483, EU074484, EU074485, GU702392, GU702394, GU702395, GU702397, HQ149713, JQ060610, JQ060611, JQ365447, JQ365448, JQ365449, JX124813, JX124814, JX185140, JX185141, JX185142, JX185143, JX185144, JX185145, JX185146, JX185147, JX185148, JX185149, JX185150, JX185151, JX185152, JX185153, JX185154, JX185155, JX185156, JX185157, JX185158, JX185159, JX185160, JX185161, JX185162, JX185163, JX185164, JX185165, JX185166, JX185167, JX185168, JX185169, JX185170, JX185171, JX185172, JX185173, JX185174, JX185175, JX185176, JX185177, MH029357, MH029362, MH029375. |
| BOLD database | *Mugil curema* | 93 | GU225396, GU225397, GU225398, GU225399, GU225400, GU440409, HM208851, HM208852, HM379821, HQ131886, HQ131887, HQ131888, HQ131892, JF911710, JQ060573, JQ060574, JQ060575, JQ060576, JQ060577, JQ060578, JQ060579, JQ060580, JQ060581, JQ060582, JQ060583, JQ060584, JQ060585, JQ060586, JQ060587, JQ060588, JQ060589, JQ060590, JQ060591, JQ060592, JQ060593, JQ060594, JQ060595, JQ060596, JQ060597, JQ060598, JQ060599, JQ060600, JQ060601, JQ060602, JQ060603, JQ060604, JQ060605, JQ060606, JQ365431, JQ365432, JQ365433, JQ365434, JQ365435, JQ365436, JQ365437, JQ365438, JQ365439, JQ365440, JQ365441, JQ365442, JQ365443, JQ840173, JQ842245, JQ842246, JQ842247, JQ842588, JQ842589, JQ842590, JQ842591, JQ842592, JQ842593, JQ842594, JX185193, JX185194, JX185195, JX185196, JX185197, JX185198, JX185200, JX185201, JX185202, JX185203, JX185204, JX185205, JX185206, JX185219, KF930147, KT075299, MF999174, MG496169, MK903544, MK903545, MK903546. |
| BOLD database | *Chelon labrosus* | 1 (outgroup) | KY176703 |
| GenBank | *Mugil cephalus* | 1 (current study) | MW584357 |

**Supplementary Table 2:** Metrics of sequence data generated on PacBio Sequel II.

| **Parameter** | **Value** |
| --- | --- |
| # subreads (>= 0 bp) | 15028480 |
| # subreads (>= 1000 bp) | 14196621 |
| # subreads (>= 5000 bp) | 11494235 |
| # subreads (>= 10000 bp) | 8841302 |
| # subreads (>= 25000 bp) | 4079726 |
| # subreads (>= 50000 bp) | 442751 |
| Total length (>= 0 bp) | 257717612248 |
| Total length (>= 1000 bp) | 257321995379 |
| Total length (>= 5000 bp) | 249407937597 |
| Total length (>= 10000 bp) | 229815154707 |
| Total length (>= 25000 bp) | 151102385623 |
| Total length (>= 50000 bp) | 26218242990 |
| # subreads | 15028480 |
| Largest subread (bp) | 282115 |
| Total length | 257717612248 |
| GC (%) | 42 |
| N50 | 28748 |
| N75 | 17157 |
| L50 | 3252517 |
| L75 | 6103971 |

**Supplementary Table 3:** Data Quality Summary of short reads Illumina sequence data used for polishing the assembly contigs.

| **Raw reads** | **Raw data (Gb)** | **Effective (%)** | **Error (%)** | **Q20 (%)** | **Q30 (%)** | **GC(%)** |
| --- | --- | --- | --- | --- | --- | --- |
| 640,674,462 | 96.1 | 99.29 | 0.03 | 97.39 | 92.86 | 41.92 |

**Supplementary Table 4:** Statistics of Hi-C raw data

| Total read pairs (RPs) analyzed | 181,042,575 |
| --- | --- |
| High quality (HQ)* RPs | 48.28% |
| HQ RPs > 10KB apart | 22.22% |
| CTGs > 10KB | 50.39% |
| Intercontig HQ RPs | 45.07% |
| Same strand HQ RPs | 38.35% |
| Split reads | 28.60% |

**Supplementary Table 5:** Quality metrics for two rounds of polishing performed for assembly contigs.

| **Polishing** | **Round 1** | **Round 2** |
| --- | --- | --- |
| Substitution errors corrected | 50528 | 10830 |
| Insertion/Deletion errors corrected | 170687 | 53867 |
| Assembly size (bp) | 643882990 | 643911503 |
| Consensus quality (%) | 99.9656 | 99.99 |

**Supplementary Table 6:** Repeat profile of *Mugil cephalus* genome assembly

| **Assembled Genome Size** | **634,849,760 bp (634627,260 bp excluding N)** | | |
| --- | --- | --- | --- |
| **Total No. of Scaffolds** | **24** | | |
| **GC Content (%)** | **41.90 %** | | |
| **Bases Masked** | **74,376,509 (11.72 %)** | | |
| **Repeat Profile** | | | |
| **Repeat class/family** | **Number of elements** | **Length occupied** | **Percentage of sequence** |
| **Retroelements** | **133451** | **23333133** | **3.68** |
| **SINEs** | **19692** | **2285123** | **0.36** |
| Penelope | 11815 | 1455496 | 0.23 |
| **LINEs** | **73955** | **15484657** | **2.44** |
| CRE/SLACS | 109 | 8554 | 0.00 |
| L2/CR1/Rex | 40922 | 9144052 | 1.44 |
| R1/LOA/Jockey | 2929 | 294363 | 0.05 |
| R2/R4/NeSL | 2716 | 811637 | 0.13 |
| RTE/Bov-B | 4644 | 1130602 | 0.18 |
| L1/CIN4 | 7446 | 2038080 | 0.32 |
| **LTR elements** | **39804** | **5563353** | **0.88** |
| BEL/Pao | 3004 | 686598 | 0.11 |
| Ty1/Copia | 6619 | 455162 | 0.07 |
| Gypsy/DIRS1 | 19472 | 3247001 | 0.51 |
| Retroviral | 7978 | 791614 | 0.12 |
| **DNA transposons** | **233659** | **33664198** | **5.30** |
| hobo-Activator | 55969 | 5992819 | 0.94 |
| Tc1-IS630-Pogo | 92564 | 19052275 | 3.00 |
| PiggyBac | 4157 | 499146 | 0.08 |
| Tourist/Harbinger | 7071 | 817318 | 0.13 |
| Other (Mirage, P-element, Transib) | 10706 | 1428900 | 0.23 |
| **Rolling-circles** | **5881** | **555616** | **0.09** |
| **Unclassified** | **1743** | **301835** | **0.05** |
| **Small RNA** | **3363** | **268065** | **0.04** |
| **Satellites** | **2879** | **362353** | **0.06** |
| **Simple repeats** | **352967** | **13966215** | **2.20** |
| **Low complexity** | **48556** | **2504867** | **0.39** |

**Supplementary Table 7:** Properties of protein-coding genes of *M. cephalus* genome assembly.

| Total sequence length, bp | 634,849,760 |
| --- | --- |
| Number of genes | 27,269 |
| Number of mRNAs | 27,269 |
| Number of exons | 220,495 |
| Number of introns | 193,226 |
| Number of CDS | 27,269 |
| Total gene length, bp | 241,612,774 |
| Total mRNA length, bp | 241,612,774 |
| Total exon length, bp | 39,056,518 |
| Total intron length, bp | 202,942,708 |
| Shortest gene, bp | 162 |
| Shortest mRNA, bp | 162 |
| Shortest CDS, bp | 153 |
| Longest gene, bp | 99,547 |
| Longest mRNA, bp | 99,547 |
| Longest exon, bp | 11,722 |
| Longest intron, bp | 84,589 |
| Longest CDS, bp | 22,095 |
| mean gene length, bp | 8,860 |
| mean mRNA length, bp | 8,860 |
| mean exon length, bp | 177 |
| mean intron length, bp | 1,050 |
| mean CDS length, bp | 1,432 |
| % of genome covered by genes | 38.1 |
| % of genome covered by CDS | 6.2 |
| mean exons per mRNA | 8 |
| mean introns per mRNA | 7 |

**Supplementary Table 8:** Non coding RNA repertoire in *M. cephalus* genome assembly.

| Row Labels | Count of target |
| --- | --- |
| 5_8S_rRNA | 5 |
| 5S_rRNA | 2954 |
| 7SK | 1 |
| ACA64 | 2 |
| ACEA_U3 | 12 |
| Antizyme_FSE | 3 |
| DLEU1_1 | 1 |
| GABA3 | 8 |
| Histone3 | 94 |
| HOTAIRM1_2 | 1 |
| IRE_I | 2 |
| IRE_II | 9 |
| K_chan_RES | 28 |
| let-7 | 22 |
| lin-4 | 5 |
| LSU_rRNA_archaea | 11 |
| LSU_rRNA_bacteria | 12 |
| LSU_rRNA_eukarya | 23 |
| mascRNA-menRNA | 1 |
| MAT2A_A | 1 |
| MAT2A_D | 1 |
| Metazoa_SRP | 17 |
| mir-1 | 4 |
| mir-10 | 12 |
| mir-101 | 2 |
| mir-103 | 3 |
| mir-122 | 1 |
| mir-124 | 5 |
| mir-126 | 1 |
| mir-128 | 2 |
| mir-129 | 4 |
| mir-130 | 1 |
| mir-1306 | 1 |
| mir-132 | 4 |
| mir-133 | 5 |
| mir-135 | 5 |
| mir-137 | 3 |
| mir-138 | 3 |
| mir-1388 | 1 |
| mir-139 | 1 |
| mir-140 | 1 |
| mir-142 | 2 |
| mir-143 | 1 |
| mir-144 | 1 |
| mir-145 | 1 |
| mir-146 | 1 |
| mir-147 | 1 |
| mir-148 | 2 |
| mir-15 | 2 |
| mir-153 | 4 |
| mir-155 | 1 |
| mir-16 | 4 |
| mir-17 | 7 |
| mir-181 | 4 |
| mir-182 | 2 |
| mir-183 | 2 |
| mir-184 | 2 |
| mir-187 | 1 |
| mir-19 | 4 |
| mir-190 | 3 |
| mir-192 | 1 |
| mir-193 | 1 |
| mir-194 | 2 |
| mir-196 | 3 |
| mir-199 | 4 |
| mir-202 | 1 |
| mir-203 | 1 |
| mir-204 | 3 |
| mir-205 | 2 |
| mir-208 | 1 |
| mir-21 | 2 |
| mir-210 | 1 |
| mir-214 | 2 |
| mir-216 | 2 |
| mir-217 | 2 |
| mir-218 | 2 |
| mir-219 | 4 |
| mir-22 | 3 |
| mir-221 | 4 |
| mir-223 | 1 |
| mir-23 | 5 |
| mir-24 | 6 |
| mir-25 | 1 |
| mir-26 | 8 |
| mir-27 | 6 |
| mir-29 | 7 |
| mir-2985-2 | 5 |
| mir-30 | 5 |
| mir-301 | 3 |
| mir-33 | 3 |
| mir-338 | 3 |
| mir-34 | 1 |
| mir-365 | 1 |
| mir-375 | 1 |
| miR-430 | 22 |
| mir-449 | 1 |
| mir-451 | 1 |
| mir-454 | 1 |
| mir-455 | 4 |
| mir-456 | 1 |
| mir-458 | 1 |
| mir-460 | 1 |
| mir-489 | 1 |
| mir-499 | 1 |
| mir-551 | 1 |
| mir-671 | 1 |
| mir-7 | 4 |
| mir-8 | 4 |
| mir-9 | 12 |
| mir-92 | 2 |
| mir-96 | 2 |
| Protozoa_SRP | 16 |
| RNase_MRP | 1 |
| RNaseP_nuc | 2 |
| SCARNA1 | 1 |
| SCARNA13 | 1 |
| SCARNA14 | 1 |
| SCARNA15 | 1 |
| SCARNA2 | 1 |
| SCARNA6 | 2 |
| SCARNA8 | 1 |
| SECIS_1 | 4 |
| Six3os1_5 | 1 |
| Six3os1_7 | 1 |
| snoMBII-202 | 2 |
| snoR38 | 1 |
| SNORA13 | 3 |
| SNORA14 | 1 |
| SNORA15 | 2 |
| SNORA16 | 1 |
| SNORA17 | 4 |
| SNORA18 | 1 |
| SNORA19 | 1 |
| SNORA2 | 1 |
| SNORA22 | 1 |
| SNORA23 | 1 |
| SNORA26 | 1 |
| SNORA29 | 1 |
| SNORA3 | 3 |
| SNORA31 | 1 |
| SNORA35 | 3 |
| SNORA44 | 1 |
| SNORA47 | 2 |
| SNORA5 | 4 |
| SNORA50 | 1 |
| SNORA53 | 2 |
| SNORA54 | 1 |
| SNORA55 | 1 |
| SNORA57 | 2 |
| SNORA62 | 1 |
| SNORA63 | 2 |
| SNORA65 | 2 |
| SNORA68 | 2 |
| SNORA70 | 2 |
| SNORA71 | 3 |
| SNORA73 | 4 |
| SNORA74 | 1 |
| SNORA75 | 1 |
| SNORA76 | 1 |
| SNORA77 | 1 |
| SNORA79 | 1 |
| SNORA8 | 1 |
| SNORA81 | 2 |
| SNORA84 | 1 |
| SNORA9 | 2 |
| SNORD10 | 2 |
| SNORD100 | 1 |
| SNORD101 | 1 |
| SNORD11B | 2 |
| SNORD12 | 4 |
| SNORD121A | 1 |
| SNORD14 | 6 |
| SNORD15 | 4 |
| SNORD16 | 2 |
| SNORD18 | 2 |
| SNORD19 | 1 |
| SNORD2 | 2 |
| SNORD22 | 1 |
| SNORD24 | 2 |
| SNORD29 | 1 |
| SNORD30 | 1 |
| SNORD31 | 2 |
| SNORD33 | 2 |
| SNORD34 | 1 |
| SNORD35 | 3 |
| SNORD36 | 2 |
| SNORD37 | 1 |
| SNORD38 | 2 |
| SNORD46 | 1 |
| SNORD47 | 1 |
| SNORD49 | 3 |
| SNORD50 | 2 |
| SNORD52 | 1 |
| SNORD53_SNORD92 | 6 |
| SNORD57 | 1 |
| SNORD58 | 11 |
| SNORD59 | 2 |
| SNORD60 | 4 |
| SNORD61 | 2 |
| SNORD65 | 3 |
| SNORD66 | 1 |
| SNORD67 | 3 |
| SNORD72 | 2 |
| SNORD73 | 1 |
| SNORD74 | 1 |
| SNORD75 | 1 |
| SNORD77 | 2 |
| SNORD79 | 1 |
| SNORD82 | 1 |
| SNORD83 | 1 |
| SNORD88 | 3 |
| SNORD89 | 1 |
| SNORD97 | 1 |
| SNORD99 | 1 |
| snosnR60_Z15 | 2 |
| snoU109 | 1 |
| snoU13 | 1 |
| snoU54 | 1 |
| snoU6-53 | 1 |
| snoU83B | 1 |
| snoU85 | 3 |
| snoZ17 | 1 |
| snoZ30 | 2 |
| SSU_rRNA_archaea | 5 |
| SSU_rRNA_bacteria | 4 |
| SSU_rRNA_eukarya | 10 |
| SSU_rRNA_microsporidia | 6 |
| Telomerase-vert | 1 |
| tRNA | 1262 |
| tRNA-Sec | 3 |
| U1 | 45 |
| U11 | 1 |
| U12 | 1 |
| U1A_PIE | 1 |
| U2 | 40 |
| U3 | 15 |
| U4 | 19 |
| U4atac | 2 |
| U5 | 12 |
| U6 | 34 |
| U6atac | 3 |
| U7 | 4 |
| U8 | 1 |
| Vault | 2 |
| Y_RNA | 2 |
| **Grand Total** | **5165** |

**Supplementary Table 9:** Synteny between *M. cephalus* and *P. haematocheila* genome assemblies.

|  | **# Blocks** | **%Coverage** | **%Double Cov** | **Inverted** | **<100kb** | **100kb - 1mb** | **1mb - 10mb** | **> 10mb** |
| --- | --- | --- | --- | --- | --- | --- | --- | --- |
| **grey mullet** | 93 | 98% | 3% | 49 | 4 | 34 | 31 | 24 |
| **redlip mullet** | 93 | 96% | 5% | 49 | 3 | 31 | 34 | 25 |
